# Aflatoxins in Feed: Types, Metabolism, Health Consequences in Swine and Mitigation Strategies

**DOI:** 10.3390/toxins14120853

**Published:** 2022-12-03

**Authors:** Roua Gabriela Popescu, Andreea Luminița Rădulescu, Sergiu Emil Georgescu, Anca Dinischiotu

**Affiliations:** Department of Biochemistry and Molecular Biology, Faculty of Biology, University of Bucharest, Splaiul Independentei No. 91–95, 050095 Bucharest, Romania

**Keywords:** mycotoxin, aflatoxin, toxicity, metabolism, swine, decontamination

## Abstract

Feeding farm animals with aflatoxin-contaminated feed can cause various severe toxic effects, leading to increased susceptibility to infectious diseases and increased mortality, weight loss, poor performance and reduced reproductive capability. Following ingestion of contaminated foodstuffs, aflatoxins are metabolized and biotransformed differently in animals. Swine metabolism is not effective in detoxifying and excreting aflatoxins, meaning the risk of aflatoxicosis is increased. Thus, it is of great importance to elucidate the metabolism and all metabolic pathways associated with this mycotoxin. The damage induced by AFB1 in cells and tissues consists of inhibition of cell proliferation, carcinogenicity, immunosuppression, mutagenicity, oxidative stress, lipid peroxidation and DNA damage, leading to pathological lesions in the liver, spleen, lymph node, kidney, uterus, heart, and lungs of swine. At present, it is a challenging task and of serious concern to completely remove aflatoxins and their metabolites from feedstuff; thus, the aim of this study was a literature review on the deleterious effects of aflatoxins on swine metabolism, as well as alternatives that contribute to the detoxification or amelioration of aflatoxin-induced effects in farm animal feed.

## 1. Introduction

**Figure 1:**
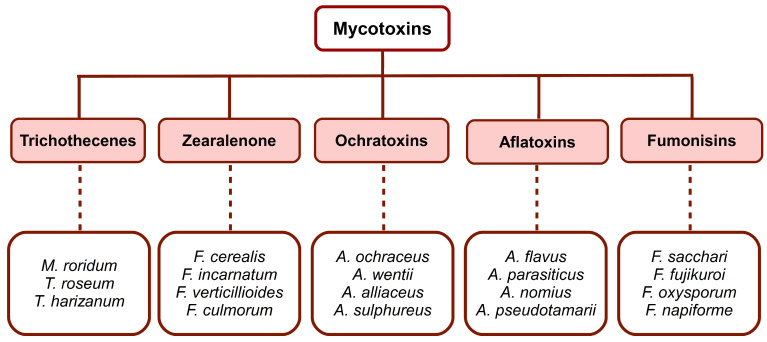
Classification of mycotoxins and the main producing species. Adapted after [8,9,10,11,12,13]. This image was made in OpenOffice Draw software.

Mycotoxins are toxins produced by certain fungal species. They are classified into five main groups (Figure 1), with specific chemical structures, that occur frequently in foods and feeds, i.e., trichothecenes, zearalenone, ochratoxins, fumonisins and aflatoxins. At the same time, fungi that produce mycotoxins are divided into two groups: those that invade before grain harvesting, a group commonly called field fungi, and those that grow only after harvesting, called storage fungi. Among the field fungi, several types of mycotoxin-producing species can be distinguished. The most important are i. *Fusarium graminearum* (deoxynivalenol, nivalenol), normally developed on the field plants; ii. *Fusarium moniliforme* (fumonisins), and sometimes *Aspergillus flavus* (aflatoxin), present in the case of senile or stressed plants; iii. *Penicillium verrucosum* (ochratoxin) and *A. flavus* (aflatoxin) that colonize the plant prior to harvesting, and subsequently predispose the crop to mycotoxin contamination. Mycotoxins are spread in animal feed, cereal crops, vegetables, and animal products. Feeding stuffs for farmed animals are considered as having the highest levels of mycotoxins [1,2,3,4,5,6].

Aflatoxins are a group of secondary metabolites that are produced by several *Aspergillus* species with increased toxicity and carcinogenic potential. Pigs, poultry and cattle are the most important farm animals affected by aflatoxicosis. The most potent toxicant is AFB1 [7].

Until 1985, the Food and Agriculture Organization reported that approximately 25% of the world’s agricultural production is contaminated with mycotoxins [14]. Taking into consideration the predicted climate change in southeastern Europe, increased cereal contamination with AFB1 and OTA is expected [15]. Contamination with aflatoxins is most predominant in the regions of Africa and Asia, due to climatic conditions that favor the development of aflatoxigenic strains in both field and storage conditions [16,17]. The risks of aflatoxin-contaminated feed depend largely on the age and physiologic status of farm animals.

The main purpose of this review is to create an overview of aflatoxin metabolism, its effects on swine health, as well as alternative procedures that contribute to the detoxification or amelioration of aflatoxin-induced effects in farm animal feed.

## 2. Types of Aflatoxins

Mycotoxins are natural compounds of low molecular weight, up to 500 Da; aflatoxins are considered the most toxic, responsible for a significant decline in agriculture. They represent the most abundant groups found in foodstuffs, oilseeds, cereals, and dairy products [6,18]. All types of aflatoxins are derived from fungal species belonging to the genus *Aspergillus* and are considered among the most harmful mycotoxins for both animals and humans [19,20,21,22,23].

Aflatoxins are colorless to pale yellow crystalline substances, freely soluble in moderately polar solvents such as chloroform, methanol, dimethyl sulfoxide, with a water solubility of 10–20 μg/mL. In conditions such as under ultraviolet light in the presence of oxygen, extremes of pH < 3 or pH > 10 and oxidizing agents, aflatoxins are unstable. For example, ammonization at high temperatures results in the opening of the lactone ring, generating the decarboxylation of an aflatoxin molecule, an irreversible reaction. Some important physical and chemical properties of aflatoxins are given in Table 1 [20,24,25,26,27,28,29].

**Figure 2:**
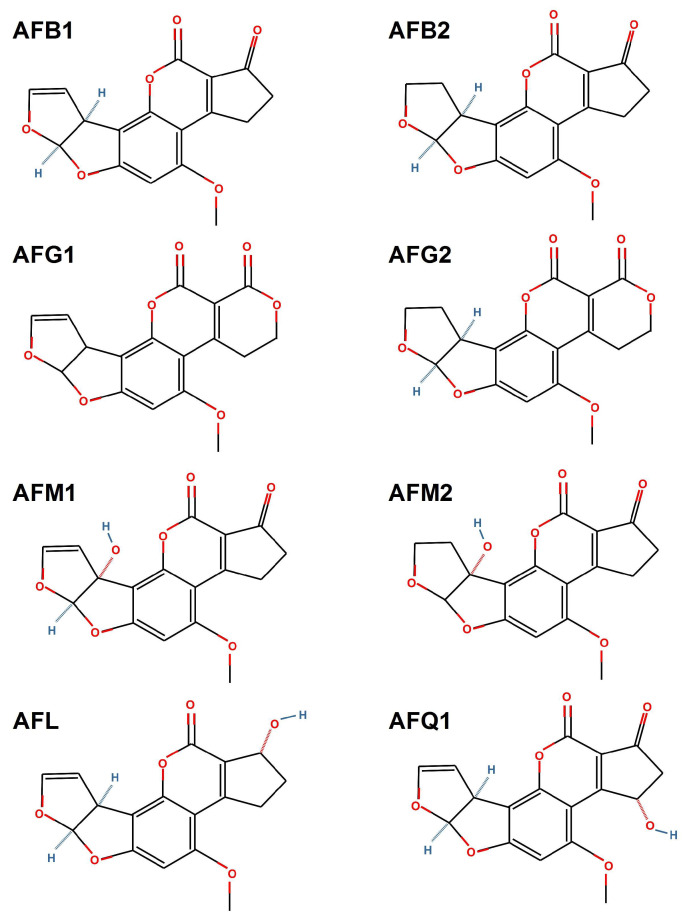
Chemical structures of aflatoxin B1 (AFB1), aflatoxin B2 (AFB2), aflatoxin G1 (AFG1), aflatoxin G2 (AFG2), aflatoxin M1 (AFM1), aflatoxin M2 (AFM2), aflatoxicol (AFL) and aflatoxin Q1 (AFQ1). This image was made in OpenOffice Draw software, v 4.1.9.

Currently, over 20 types of aflatoxins are known and among the best known are B1, B2, G1, G2, M1, M2, aflatoxicol and aflatoxin Q1 (Figure 2). Some of these forms are derivatives or metabolites of animal metabolism. For example, aflatoxin M1 and aflatoxin M2 are the metabolites of aflatoxin B1 and aflatoxin B2 which are found in the milk of lactating mammals fed with aflatoxin-contaminated feed [20,29,30].

**Table 1:** Physical and chemical properties of major aflatoxins. Adapted after [29,31,32,33,34].

Aflatoxin Type	Molecular Formula	Molecular Weight (g /mol)	Melting Point (°C)	Fluorescence	
				λ Excitation (nm)	λ Emission (nm)
B1 [29]	C_17_H_12_O_6_	312	268–269	223	425
B2 [29]	C_17_H_14_O_6_	314	286–289	265	425
G1 [29]	C_17_H_12_O_7_	328	244–246	243	450
G2 [29]	C_17_H_14_O_7_	330	237–240	265	450
M1 [33]	C_17_H_12_O_7_	328	299	365	435
M2 [34]	C_17_H_14_O_7_	330	293	360	450
Aflatoxicol [32]	C_17_H_14_O_6_	314	225	325	425
Aflatoxin Q1 [31]	C_17_H_12_O_7_	328	250	365	466

### 2.1. Aflatoxins B1 and B2

Aflatoxin B1 (AFB1) is the most potent carcinogenic mycotoxin naturally produced by Aspergillus species such as A. flavus, A. parasiticus, A. nomius, A. bombycis, A. arachidicola, A. minisclerotigenes, A. ochraceoroseus, A. pseudotamarii and A. rambellii, and it exerts harmful effects on humans and animals. The sensitivity degree and toxicity of AFB1 vary significantly between species, due to differences in its biotransformation. Some animals are considered extremely susceptible to AFB1, especially turkeys, rats, pigs, sheep, and dogs, whereas others such as monkeys, mice and chickens are considered resistant. The LD_50_ values for aflatoxin B1 are variable, depending on species and sex, with values ranging from 9 to 60 mg of AFB1 per kg of body weight [20,30,35,36,37,38].

Aflatoxin B2 (AFB2) is a blue-fluorescent, toxic secondary metabolite produced by the same species as AFB1, such as *A. arachidicola, A. flavus, A. minisclerotigenes, A. nomius* and *A. parasiticus*. This metabolite can be synthesized through multiple sequences that begin with a [2+3]-cycloaddition between quinone and 2,3-dihydrofuran [20,39,40,41].

### 2.2. Aflatoxins G1 and G2

Aflatoxin G1 (AFG1) and aflatoxin G2 (AFG2) are toxins produced by species of the common soil fungi, *A. parasiticus, A. nominus, A. bombyccis, A. arachidicola* and *A. flavus*. The presence of AFG1 is associated with toxicity and hepato-carcinogenicity in human and animal populations, while AFG2 has much lower activity [20,30,42,43].

### 2.3. Aflatoxins M1 and M2

The aflatoxins M1 (AFM1) and M2 (AFM2) are mammalian bio-conversion products or 4-hydroxy derivatives of AFB1 and AFB2, respectively, produced by *A. flavus* and *A. parasiticus*. After entering the body of humans or animals, AFB1 and AFB2 are metabolized by the hepatic microsomal mixed function oxidase system (cytochrome P450) to a reactive epoxide intermediate, but they can be also hydroxylated to the less harmful aflatoxins M1 and M2. In the case of an animal that ingests feed contaminated with AFB1, a percentage between 0.5% and 5% of the toxin ingested is biotransformed in the liver into AFM1. Milk, cheese, and other dairy products contain residues of AFM1 and AFM2 that should not exceed the limit of 50 ng per kg in Europe, 500 ng per kg in the USA, and 100 ng per kg in Iran [20,23,30,44,45,46,47] for human consumption.

### 2.4. Aflatoxicol

The first report on natural contamination of food with aflatoxicol (AFL) appeared in 1984 [48]. AFL is one of the metabolites of AFB1, formed by the selective reduction of cyclopentanone carbonyl of AFB1, and has two stereoisomers (AFL1 /AFL-A /Ro and AFL2 or AFL-B) which differ by the orientation of the hydroxyl group in the cyclopentene ring. Both AFL forms are produced by the biological reduction catalyzed by enzymes present in fungi, such as: *Tetrahymena pyriformis, Trichoderma viride, Dactylium dendroides, Streptococcus lactis, Absidia repens, Mucor griseocyanus, Aspergillus niger, Mucor ambiguus, Tetrahymena pyriformis* and *Rhizopus spp*. Although AFL is eighteen times less toxic than AFB1, it was shown that AFL is carcinogenic and a potent frameshift mutagen [32,49,50,51,52].

### 2.5. Aflatoxin Q1

Aflatoxin Q1 (AFQ1) is a monohydroxylated derivative of AFB1, being one of the major AFB1 metabolites which appear after incubation of microsomal fraction from the mammalian liver with AFB1. The microsomal fraction is rich in CYP3A4 and other CYP450 enzymes which are responsible for the activation of AFB1 into the epoxide form, and for conversion into a less toxic detoxification metabolite, AFQ1. Initially it was found in the urine of rhesus monkeys orally exposed to AFB1. On the other hand, Yourtee et al. [53] showed that AFQ1 might be a major metabolite in the detoxification pathway of the native mycotoxin. AFQ1 is approximately eighteen times less toxic and approximately eighty-three times less mutagenic than AFB1 [30,53,54,55].

## 3. Aflatoxins’ Metabolism: Biochemical, Molecular and Cell Signaling Aspects

**Figure 3:**
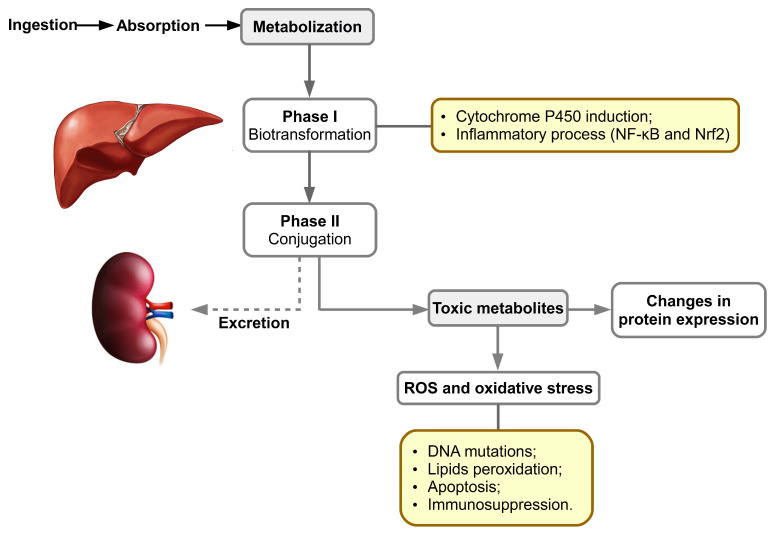
The adverse cellular effects of mycotoxins and their metabolites. Adapted after [56,59,60,61]. This image was made in OpenOffice Draw software, v 4.1.9.

After ingestion of contaminated food, aflatoxins are absorbed in the intestine; following their distribution, metabolism and excretion, the liver is the first and main organ affected (Figure 3). They also accumulate in muscle. P450 cytochromes play an important role in phase I biotransformation of xenobiotics, especially those belonging to families 1 and 3 [56]. In mammals, the enzymes with the highest levels of protein expression, and involved in the conversion of aflatoxins, are CYP1A2 and CYP3A4. The metabolite resulting from the oxidation reaction can bind to DNA, causing genotoxicity, and proteins generating cytotoxicity. For example, AFB1 binds to guanine residues of nucleic acids, resulting in AFB1 adducts that can lead to transversion of guanine–cytosine (GC) to thymine–adenine (TA) and implicitly to irreversible DNA damage. Binding of AFB1 to proteins is irreversible, the most well-known adduct being ADB1-lysine in albumin. In the first stage of metabolic oxidation in the liver, an epoxy reactive intermediate (e.g., AFB1-8,9-epoxide) is formed or this is hydrolyzed to a less toxic form, AFM1 [57,58]. 

The cytochrome P450 superfamily consists of enzymes involved in xenobiotic metabolism and endogenous compound oxidation; thus, Phase I enzymes catalyze the reactions of hydroxylation, sulphoxidation, epoxidation, N-, O- and S-dealkylation, oxidative aromatic hydroxylation, desulfuration, denitrosation, and dehalogenation aiming for the addition of functional polar group(s). In porcine hepatic tissue, the CYP450 proteins expressed are represented by CYP2A19 (34%), CYP2D25 (25,5%), CYP2C49 (11.2%), CYP2E1 (8.1%), CYP3A39 (8,1%), CYP3A29 (5,8%), CYP2C33 (5%) and CYP1A2 (2.3% of the total liver CYPs, respectively) [62,63,64,65,66,67,68].

Phase II of metabolism implicates conjugation reactions of metabolites previously formed [69] with glucuronic acid and sulfate especially. Subsequently, the epoxide metabolite generated in phase I may be detoxified in phase II by glutathione conjugation, through hydrolysis by an epoxide hydrolase to AFB1-8,9-dihydrodiol, or by reduction to a less toxic metabolite such as AFM1 or AFQ1 [43,70,71,72,73]. The resulting metabolites are excreted through the biliary pathway, followed by the urinary pathway.

By RNA-seq technology it was proved that in vitro exposure of bovine fetal hepatocyte cell line (BFH12) to AFB1 affected the cells` transcriptome. Gap junction protein beta 2 and Follistatin genes—the latter being involved in proliferation and colony expansion of progenitor populations of hepatocytes—as well as those of ornithine decarboxylase and A-Raf proto-oncogene have been upregulated. Instead, genes that codify for tumor suppressors, such as those of collagen type XVIII alpha 1 chain (COL18A1), collagen type 1 alpha 2 chain (COL1A2), as well as that for natriuretic peptide receptor 3 have been downregulated. The treatment with this mycotoxin also upregulated the following CYP isoforms: CYP26B1, CYP3A4, CYP27B1 and downregulated CYP1A1, CYP1B1, CYP19A1, CYP36A1, CYP4B1 [74].

The same study from Pauletto et al. [74] revealed that all analyzed glutathione-S-transferase genes, except those for omega 1 and pi1 isoforms, have been downregulated. The gene sets for TNF-α signaling via NF-kB, oxidative phosphorylation, DNA repair, inflammatory response, KRAS signaling, p53 pathway, PI3K-Akt-mTOR signaling, apoptosis and hypoxia have been upregulated by AFB1 treatment of BFH12 cells. In the same conditions, other gene sets for epithelial–mesenchymal transition, bile acid metabolism, estrogen response and heme metabolism have been downregulated.

Recently, based on transcriptomic data and post translational analyses, it was postulated that Toll-Like Receptor (TLR2) activation is involved in AFB1-induced inflammation and oxidative stress in BFH12 cells [75]. Moreover, in a chicken hepatocarcinoma cell line (LMH) exposed to AFB1 differentially, expression analysis revealed that 1006 genes have been upregulated and 791 downregulated, compared with the control treatment. The mRNA expression of CYP27A1, CYP1A4, FABP2, PPARα and GSTT1 were significantly decreased by this mycotoxin treatment, whereas genes responsible for focal adhesion and MAPK pathways were upregulated compared with control ones [76].

Previously it was noticed that in HepG2 cells treated with AFB1, increases in the expressions of miR-34A and miR-33a-5p led to an important decrease of β-catenin, c-myc and cyclin D1 levels in the Wnt signaling pathway, generating an important risk of hepatocellular carcinoma [77,78]. The exposure to this mycotoxin also inhibits protein synthesis and due to this, enzymes` levels of different metabolic pathways are affected [79].

Recently, it was proved that AFB1 exposure of cells affects the respiratory chain, generating reactive oxygen species (ROS). If these are not counteracted by the antioxidant enzymatic and non-enzymatic systems, oxidative stress occurs [80]. The excess of ROS attacks polyunsaturated fatty acids from glycerophospholipids, generating end products of lipid peroxidation, as well as DNA and proteins. Lipid peroxidation and oxidative damage to DNA play a major role in the toxicity of aflatoxins. 

## 4. Aflatoxin’s Toxicity in Swine

The monogastric animals are more susceptible to AFB1, compared to the ruminants, since bacteria from the rumen section of the stomach can metabolize mycotoxins [81]. The pig’s caloric need is supplied by carbohydrates and fats in great extent. Cereal grains represent a source of carbohydrates, and they are included in swine ratio in up to 85% of the ingredients [82]. Maize, wheat, barley and oat are used in pigs’ nutrition, and represent a common source of mycotoxins in feed.

In recent years, the domestic production and industrial swine industry have been heavily affected by viral infections, such as African swine fever and mycotoxin contamination [83,84]. Mycotoxins with the greatest economic impact on swine breeders are aflatoxins, zearalenone, deoxynivalenol, trichothecenes, T-2 toxin and ochratoxins [85,86,87]. Growing pigs are highly susceptible to mycotoxins. One of the main difficulties encountered in controlling mycotoxins is that more than one type of mycotoxin is present in a batch of fodder, or cereal for pigs, at the same time. Thus, feeding pigs contaminated feed with several types of mycotoxins, even if they are in concentrations at the minimum recommended by the European Union, can cause numerous negative effects in animals due to cumulative toxic effects. The most common symptoms of mycotoxicosis in swine are the refusal to eat, decrease in growth rate, reproductive disorders and decreased immune status [88,89,90,91,92]. In the process of breeding and producing pigs about 60–70% of the costs are due to feed [93]. 

Luthy et al. [94] showed that in pigs approximately 20% of the radioactive AFB1 dose was excreted in the urine during nine days. AFM1, the metabolite of AFB1, was found in the range of 80–420 pg/mL in the urine of pigs fed with 26.48 µg AFB1/kg of body weight for 42 days [94,95,96,97,98,99].

Exposure of swine to aflatoxins can cause a variety of chronic or acute syndromes depending on the type of aflatoxin and level of consumption; aflatoxins can generate increased susceptibility to infectious diseases and increased mortality, weight loss and poor performance, reduced reproductive capability, changes in clinical biochemical patterns, and suppressed immune function [100]. 

The maximum tolerable levels of aflatoxins in pig diets depend on age. According to the FDA, the regulatory limits for swine aflatoxin B1 are <20 ppb for piglets, <100 ppb for specimens used for reproduction and <200 ppb AFB1 for those in the finishing period [101,102]. Aflatoxin exposure generates in pigs: a low growth rate, poor conversion of food, increased mortality, impaired coagulation of blood and kidney function, changes in the immune response, increased susceptibility to disease and decreased resistance to stress [103,104]. 

The liver is the organ most affected by the ingestion of aflatoxins because it receives and concentrates all compounds carried by the bloodstream. Extremely high concentrations of aflatoxins (over 1000 ppb, previously reported in Ugandan crops, mainly maize, peanuts and cassava) cause hepatitis, hepatic necrosis, increased clotting time, and finally the death of animals caused by severe hemorrhage. In a lighter, subacute form, aflatoxicosis causes hepatic lipidosis, portal fibrosis and liver tumors [105,106]. 

Additionally, the resulting aflatoxin metabolites can be transmitted from lactating sows to nursing pigs, via milk, consequently contaminating the piglets which are more sensitive to stunted growth; thus, this may cause up to a 20% mortality in piglets, characterized by enterocolitis, diarrhea, and a suppressed immune system which leads to decreased resistance to infectious diseases. Prodanov-Radulović et al. [91] reported the presence of AFM1 in the milk of nursing sows consuming diets containing AFB1. Furthermore, Weaver [107] showed that the concentration of AFM1 was about 1.7 times higher in colostrum, than milk of nursing sows, because AFM1 binds to milk casein and therefore is transferred to the piglets [29,72,91,108,109,110,111].

The diseases caused by the consumption of aflatoxins are known as aflatoxicosis. Swine metabolism is not effective in detoxifying and excreting aflatoxins, which increases the risk of aflatoxicosis. The main biological effects of aflatoxins in suckling piglets, growing, and finished and breeding pigs are carcinogenicity, immunosuppression, mutagenicity, teratogenicity, decreased feed efficiency and poor weight gain, impaired liver and altered serum biochemical parameters. Severe effects in swine can lead to acute hepatitis, systemic hemorrhages, nephrosis and death [71,72,112]. Some authors have shown that swine fed with low levels of aflatoxins presented signs of pulmonary edema, reduced feed consumption and body weight gain, as well as a decrease in the activity of enzymes that catalyze the oxidative decarboxylation, total serum proteins, total leukocyte count and blood pressure [72,113,114,115].

Another toxic effect in swine exposed to aflatoxins is the alteration of the inflammatory response, known as immunotoxicity. In weanling pigs fed for 28 days with low doses of aflatoxins [116], reduced synthesis of pro-inflammatory cytokines and an increase in anti-inflammatory ones were noticed. 

Immunomodulatory effects of AFB1 have also been proven in swine. Studies conducted by Meissonnier et al. highlighted impaired lymphocyte activation and increased cytokine expression (TNF-α, IL-1β, IL-6, IL-10, and IFN-γ) in pigs vaccinated with ovalbumin, after dietary AFB1 exposure [117,118]. In contrast, Marin et al. [118] showed that aflatoxins did not exert any effect on regulatory cytokines produced by either the Th1 (IL-2) or the Th2 (IL-4) subset of lymphocytes.

AFB1 is a very strong inhibitor of lymphocyte proliferation. Stec et al. [119] showed that a concentration of 0.02 µg AFB1/mL reduced up to 50% of lymphocytes, isolated from peripheral blood taken from 7-week-old pigs after a 72-hour exposure period, suggesting that AFB1 is a very strong inhibitor of in vitro lymphocyte proliferation in pigs [116,119,120].

The effects on sperm motility or on the reproductive performance of gilts depend on aflatoxin doses also. The maturation rates of oocytes decreased significantly in the case of acute exposure to 50 μM AFB1, probably because most oocytes have been arrested at the germinal vesicle breakdown or meiosis I stage, resulting in early oocyte apoptosis and increased Bak, Bax, Bcl-xl mRNA levels. This could suggest that AFB1 disrupts porcine oocyte maturation through the modulation of epigenetic modifications, oxidative stress, excessive autophagy and apoptosis [121,122].

In summary, aflatoxins induce pathological lesions in the liver, spleen, lymph node, kidney, uterus, heart and lungs of swine. Severe toxicity causes collapse and death within several hours, acute toxicity causes death within 12 h, and with subacute toxicity death occurs after about 20 days [123,124,125,126,127,128].

## 5. Methods to Reduce Aflatoxins’ Toxicity

The need for solutions to ameliorate the effects of mycotoxins on food-producing swine prompts increased research in this area. Currently, there are few national and international studies that focus on the effects of aflatoxins at the hepato-nephrotoxic level in swine. Considering this, studies regarding detoxification methods and the influence of certain feed additives on the toxicity of aflatoxins, in swine liver and kidneys, are of great importance. 

Aflatoxin decontamination procedures have been developed to inactivate or remove it from feed stuffs, without leaving any chemical residues. These must be cost-effective to keep the final market price reasonable. The methods used for decontamination of aflatoxins can be divided into biological, chemical and physical methods. All these methods must ensure that the degradation process maintains the nutritive value of feed and will not introduce one or more toxic substances. Prevention is the most desirable method of reducing aflatoxin contamination but needs much more improvement in terms of agricultural storage methods, practices in harvesting and handling of crops. Therefore, the recognition of problems caused by mycotoxins in food and feed is the first step to prevention, which will allow farmers to produce good quality food for the animals [29,72,129,130,131].

The general chemical methods used against aflatoxins are based on chemical agents that deactivate and degrade aflatoxins by oxidation and/or hydrolysis of the lactone ring from the polyketide backbone of aflatoxins, or by oxidation of the double bond of the terminal furan ring. However, the use of these agents is limited due to the problems associated with their residues [29,131,132].

Physical methods involve the separation of contaminated fractions, removal, or inactivation of aflatoxins by physical means, such as heating, cooking, roasting, and radiation. Due to the limited solubility of aflatoxins in water, these procedures are regarded as being unfeasible and economically inefficient. Therefore, decontaminating products contaminated with mold requires a multi-step process that involves mechanical sorting and washing. Jalili [133] mentioned that processing methods such as boiling, roasting, baking, and steaming in maize products destroyed aflatoxins to a considerable extent of 50–70%. 

Adsorption is another physical method for aflatoxin decontamination and involves the binding of a toxic compound, to the adsorbent compound, during digestion in the gastrointestinal tract of farm animals. Examples of adsorbents are active carbon, diatomaceous earth, alumina clay, alumina bentonite, montmorillonite; sodium and calcium aluminum silicates, mainly zeolite; phyllosilicates and hydrated sodium calcium aluminosilicate; complex carbohydrates such as cellulose, and the polysaccharides present at the cellular walls of yeasts and bacteria e.g., glucomannans and peptidoglycans; and synthetic polymers of cholestyramine, polyvinyl pyrrolidone, and its derivatives [29,131,133,134].

Efficient drying of farmed feed is an effective measure against fungal growth and aflatoxin production. The correct way of drying is the best manner of avoiding fungal growth and mycotoxin production in grain after harvest. When natural drying in the sun is not possible, most of the time because climate conditions do not allow this, in order to reduce or prevent the production of most mycotoxins, drying should take place as soon as possible after harvest, and as rapidly as feasible, or otherwise it is necessary to use a form of mechanical drying [129,135].

Biological decontamination of aflatoxins is another strategy in which the degradation is achieved, by using modified strains of *Aspergillus* to reduce aflatoxin contamination by competitive inhibition, or by using genetically modified plants. For example, in Africa, Central America and Asia, the populations experience high levels of exposure to dietary aflatoxin from maize, which is an important part of the human diet in these locations. One of the strategies used in these regions involves the use of transgenic maize (Bt corn) in order to control mycotoxin contamination. The second approach involves the use of a food supplement (NovaSil clay) in order to absorb aflatoxins in the gastrointestinal tract and, therefore, reduce the toxin bioavailability. The third method is based on a modified strain of *A. flavus* that does not produce aflatoxins. Another alternative for biological decontamination is the addition of antioxidant compounds in animal feed, in order to reduce the toxic effects of aflatoxins, or to inhibit the growth of aflatoxin-producing fungal species. Examples of antioxidant compounds are chlorophyll and its derivatives, selenium, medicinal herbs and plant extracts [71,136,137].

The presence of polyphenolic compounds in feed, especially representatives of the flavonoid group, can attenuate the mycotoxin-induced inflammatory process by modulating the activities of NF-κB and Nrf2 [138,139]. However, the potential anti-inflammatory effects of polyphenols have, so far, been less investigated in farm animals.

Currently, wine production is one of the main agricultural activities around the world, which is accompanied by the generation of large amounts of waste and by-products rich in antioxidant compounds [140]. Examples of such compounds are stilbens (resveratrol), anthocyans, flavones, flavonones and isoflavones [59].

A strategy for reducing exposure to mycotoxins in animals includes supplementation of feed products with detoxifying additives, which allows for counteraction of their toxic effects [29,141,142,143,144].

## 6. Conclusions

Aflatoxins produced by various fungi during the pre- and post-harvest stages of various food and feed, cause adverse effects in different animals and negative economic impacts worldwide. Significant advances have been achieved in our understanding of aflatoxins’ metabolism. Swine are particularly sensitive to aflatoxin exposure due to ineffective detoxification and excretion. The major challenge of ongoing and future research will remain the identification of members of metabolic pathways that link aflatoxin toxicity in swine, to the perturbations of cell metabolism and oxidative stress. Current methods cannot completely remove aflatoxin metabolites from swine diets. Therefore, it is desirable to prevent the contamination of feed by aflatoxins, which is achievable by using different procedures, including feed storage in dry areas and improved management techniques, in order to develop strategies that contribute to the detoxification or amelioration of aflatoxin-induced effects in farm animals, in an efficient and cost-effective manner.

## Data Availability

Not applicable.
